# Underpinnings of the Halachic Approach to BRCA Screening and Intervention: Facilitating Provider Counseling for Observant Jewish Populations

**DOI:** 10.5041/RMMJ.10522

**Published:** 2024-04-28

**Authors:** Chaya Greenberger, Pnina Mor

**Affiliations:** 1 Adjunct Professor of Nursing, Lev Academic Center, Jerusalem, Israel; 2 Medical Genetics Institute, Shaare Zedek Medical Center, Jerusalem, Israel

**Keywords:** BRCA mutations, screening interventions, halachic approach, health provider, decision-making

## Abstract

**Background:**

Halacha is the corpus of Jewish law which serves as a life blueprint for observant Jewish individuals. Health professionals counseling halachically observant populations at risk for breast cancer gene (BRCA) mutations should be well informed of the halachic approach to screening for BRCA mutations and subsequent interventions.

**Aim:**

To address the intersection of halacha with ethical norms and current medical evidence-based data as they relate to potential and identified BRCA mutation carriers at their various stages of decision-making.

**Results:**

Halacha, ethics, and medicine have much in common, but there are specific principles which guide halacha; decision-making in light of halacha is complex and varies with respect to the multi-faceted aspects of screening and intervention. Halacha encourages the exercise of autonomy regarding situations in which beneficence is not clear-cut and dependent on subjective perceptions.

**Conclusions:**

Health professionals knowledgeable of halacha are better equipped to counsel the observant Jewish population at risk of BRCA mutations or identified as mutation carriers, enabling them to present targeted questions to halachic authorities and thus achieve optimal decision-making.

## INTRODUCTION

The population at risk for breast cancer gene (BRCA) mutations has both ethical and medical issues to consider with respect to screening uptake and subsequent interventions. The observant Jewish population is unique in that it additionally considers halachic issues. The health professional plays a key role as counselor and facilitator and is often the first authority encountered by individuals, including those who are observant, as they embark on the process of BRCA decision-making. The aim of our discourse is to deepen the understanding of health professionals on how halacha impacts the decision-making process of the observant Jewish BRCA mutation carrier. This aim necessitates engaging with sources of biblical and post-biblical origin; the latter, an interpretation and expansion upon the Bible, incorporate, among other works, the Talmud, Halachic Codes such as *Yad HaChazakah* (Maimonides) and *Shulchan Aruch* (Rabbi Joseph Karo), and Responsa literature up to the present time. As an integral unit, these writings are often referred to as the Torah.

## SCIENTIFIC BACKGROUND

Advances in human genetics provide a better understanding of disease biology, enable more specific characterization of malignancies, and facilitate development of new therapeutic modalities.^1^ Genetic testing enables providers and clients to make more informed health-care decisions.^2^

Research has identified two autosomal, dominantly inherited tumor suppressor genes, BRCA1 and BRCA2, which are linked to hereditary breast and ovarian cancers.^3^,^4^ The BRCA1 gene and the BRCA2 gene are found on the long arm of chromosomes 17 and 13, respectively. Everyone has two copies of each of these genes, one from each parent. These genes produce proteins that prevent abnormal cell growth that can result in cancer.^5^ When there is a mutation, protein production is suppressed, and abnormal cell growth is more likely. People who inherit harmful variants in one of these genes have increased risks of several cancers—most notably breast and ovarian cancer. Approximately 1 in 40 individuals of European Ashkenazi Jewish descent was shown to carry a BRCA mutation.^6^,^7^ The presence of a gene mutation does not mean that a person will invariably develop cancer.^8^ However, research indicates that individuals who carry this genetic mutation are at greatly increased risk of developing active disease. People who have inherited a harmful variant in BRCA1 or BRCA2 also tend to develop cancer at younger ages than people who do not have such a variant.^9^ Overall, only 5%–10% of all breast and ovarian cancers are the result of BRCA mutations.^10^,^11^

About 13% of women in the general population will develop breast cancer sometime during their lives. By contrast the risk for breast cancer for BRCA1/2 mutation carriers by 70–80 years of age is roughly five to seven times higher (55%–72% of women who inherit a harmful BRCA1 variant and 45%–69% of women who inherit a harmful BRCA2 variant). About 1.2% of women in the general population will develop ovarian cancer sometime during their lives.^12^,^13^ By contrast, 39%–44% of women who inherit a BRCA1 mutation and 11%–17% of women who inherit a BRCA2 mutation will develop ovarian cancer by 70–80 years of age. Kuchenbaecker and the BRCA1 and BRCA2 Cohort Consortium^9^ reported in their study that the cumulative risk of developing breast cancer by age 80 years is 72% for BRCA1 mutation carriers and 69% for BRCA2 mutation carriers. For ovarian cancer, the cumulative risks by age 80 years are 44% for BRCA1 mutation carriers and 17% for BRCA2 mutation carriers. Breast cancer incidence for mutation carriers increases with age in early adulthood then plateaus to remain relatively constant throughout their remaining lifetime. The age at which this plateau was reached was 31–40 years for BRCA1 carriers and 5–10 years later for BRCA2 carriers. The incidence during the plateau was similar for both groups of mutation carriers.^9^ The BRCA mutations are also linked to other gynecological cancers, although the lifetime risks are not as high. These cancers include serous tubal intraepithelial carcinoma,^14^ primary peritoneal carcinoma,^15^,^16^ and serous and/or serous-like endometrial cancer.^17^,^18^ Men with BRCA mutations are also at increased risk of breast cancer and prostate cancer,^19^,^20^ while both men and women with BRCA mutations have an increased risk of pancreatic, melanoma, stomach, and colon cancers.^15^,^21^ Discussion regarding these cancers is beyond the scope of this paper.

**GLOSSARY**

Aguna:
A woman whose husband is missing or refuses to grant her a religious divorce. She is literally “chained” as she cannot remarry.
Babylonian Talmud:
The most basic component of the Oral Torah, consisting of 36 tractates, which interprets and expounds upon the Hebrew Bible. It is composed of the Mishna, codified in 200 CE, and the Gemara (also referred as the Talmud) written and compiled in Babylonia, codified in 500 CE. It contains both legal and narrative texts and forms the underpinning for all subsequent halachic as well as narrative works.
Halacha:
The body of Jewish laws, customs, and traditions which serves as a blueprint for the religious life of observant Jewish individuals. It is derived from the collective corpus of both the written Torah (i.e. the five books of Moses) and the Oral Torah (i.e. the Talmud and subsequent codes of Jewish law and the Responsa literature throughout the ages).
Pikuach nefesh:
Literally, “vigilance with respect to life,” it denotes the obligation to override virtually all commandments in order to save a life.
Responsa:
Compilations of questions posed to halachic experts and their respective answers. As a rule, each expert takes account of precedent, the specifics of the particular question presented, as well as the context of Jewish life, in coming to his decision. Responsa literature is a continuum.
Shulchan Aruch:
The most widely accepted code of law for Jewish practice, authored in 1563 CE by the Sephardic Rabbi Joseph Karo and glossed by Rabbi Moshe Iserles who added the Ashkenazic tradition. There are numerous halachic experts who have, over the generations, expanded on this code.
Torah:
Technically the five books of Moses, but often used in its broader sense as including all the 24 books of the canonized Hebrew Bible and in many cases as a reference to the totality of the teachings derived from both the written scriptures and the Oral Torah over time.
Yad HaChazakah:
The comprehensive halachic code written by Maimonides between 1170 and 1180, also known as *Mishneh Torah*, organized topically and serving as a basis for later codes.

The current clinical model for referral to genetic testing is based on personal or family history of breast, ovarian, tubal, or peritoneal cancer or those who have an ancestry associated with BRCA1/2 gene mutations.^8^,^22^ The standard approach requires individuals to be aware of their family history of cancer, understand its importance, seek genetic counseling, and, when recommended, go ahead with genetic testing. Population testing for all Jewish Ashkenazi women has been considerably assessed, demonstrated to be acceptable, safe, and effective, and can be undertaken in community health-care settings. It has the capability to identify >50% additional BRCA carriers who would have been missed by conventional clinical criteria, and thus has the potential to save lives. Population-based BRCA testing in the Jewish population is acceptable in many countries, offering non-affected healthy BRCA mutation carriers the opportunity to be proactive about intensive surveillance or risk-reducing surgeries.^23^

On receipt of a positive test for BRCA mutation, the healthy carrier is further informed of the strategies available to maintain her health. International organizations (National Comprehensive Cancer Network, European Society for Medical Oncology, Society of Obstetricians and Gynecologists) provide comprehensive guidelines.^22^,^24^,^25^ The breast cancer screening/surveillance guidelines for the healthy BRCA carrier include: breast awareness starting at age 18 years, clinical breast exam every 6–12 months starting at age 25 years, breast screening with annual magnetic resonance imaging (MRI) of the breast from age 25–29 years, annual mammogram from age 30. Over age 75 management should be considered on an individual basis. For BRCA mutation carriers who do not have active breast cancer but have been treated for breast cancer in the past and have not undergone a bilateral mastectomy, screening should continue, with annual breast MRI and mammogram similar to the healthy BRCA mutation carrier. The guidelines recommend discussing the option of risk-reducing bilateral mastectomy and considering the option of risk reduction agents such as tamoxifen. The ovarian cancer risk guidelines recommend risk-reducing salpingectomy-oophorectomy (RRSO) for BRCA1 mutation carriers between the ages 35 and 40 years, or on completion of childbearing years. In women with BRCA2 mutations, ovarian cancer onset is an average of 8–10 years later, therefore risk-reducing surgery is delayed until age 40–45 years.^22^,^24^,^25^ In addition it is recommended to discuss with women of childbearing ages the option of stopping the chain of BRCA heritage with *in vitro* fertilization and preimplantation genetic testing.^22^

The use of MRI and mammography/ultrasound in breast cancer screening is substantially reliable if there is compliance. In situations where compliance with breast cancer screening is lacking, this would perhaps tip the balance in favor of surgical intervention. Studies have reported that anxiety levels may peak during times of surveillance until negative results are received.^26^ Tumor marker CA125 and transvaginal ultrasound recommended in the past for ovarian cancer screening are not reliable and have not been proven statistically significant. They are therefore not recommended in the current guidelines.^22^

### Risk-reducing Breast Surgeries

Risk-reducing surgery is an alternative to the rigid breast imaging screening protocol recommended to BRCA mutation carriers. The decision to undergo risk-reducing bilateral mastectomy (RRBM) is the woman’s choice and will reduce her risk of breast cancer by 90%–95%.^27^ Risk-reducing surgery involves removing as much of the “at-risk” breast tissue as possible. Risk-reducing surgery does not guarantee that cancer will not develop because not all at-risk tissue can be removed by these procedures. The current guidelines recommend that counseling a BRCA mutation carrier should include a discussion regarding the option of RRBM, the degree of protection it provides, the reconstruction options, and risks. The discussion should address psychosocial and quality-of-life aspects of undergoing RRBM.^22^ In addition, the family breast cancer history and residual breast cancer risk should be considered during counseling, taking into account age and life expectancy. Metcalfe et al. examined mortality rates after RRBM. Her group compared BRCA1/2 mutation carriers who chose RRBM versus those who preserved their breasts and concluded that, in women with a BRCA1 or BRCA2 pathogenic variant, RRBM reduced the risk of breast cancer (HR 0.20). The probability of dying of breast cancer within 15 years after RRBM was less than 1% (0.95%).^27^

The Cochrane report of 2019 summarizes numerous studies analyzing RRBM. The report concludes that RRBM reduces the incidence of breast cancer and/or the number of deaths or both.^28^ Heemskerk-Gerritsen and colleagues concluded from their study that RRBM was associated with lower mortality than surveillance for BRCA1 mutation carriers at the age of 65. The probability of not having died due to breast cancer was 99.7% for the RRBM BRCA1 group and 93% for the surveillance group. For BRCA2 mutation carriers, RRBM may lead to similar breast cancer-specific survival as surveillance rates, with 100% in the RRBM and 98% in the surveillance group; therefore discussion regarding RRBM for BRCA2 mutation carriers should be considered according to personal family history.^29^ In women who have had cancer in one breast, contralateral risk-reducing mastectomy (removing the other breast) may reduce the incidence of cancer in the healthy breast, but there is insufficient evidence that this improves survival because of the continuing risk of recurrence or metastases from the original cancer. The recommendation to undergo RRBM, in contrast to risk-reducing salpingectomy-oophorectomy, is not age-dependent, although superior cosmetic results are reported in younger women (30–40 years) when considering reconstruction.^22^

In general, most women are satisfied with their decision to undergo RRBM and report reduced anxiety and worry of developing and dying from breast cancer. Although satisfied with the decision to undergo RRBM, some women report experiencing less satisfaction with the cosmetic results, body image, and sexual feelings.^30^

### Risk-Reducing Bilateral Salpingectomy-Oophorectomy; Surgically Induced Menopause and Hormone Replacement Therapy

The absence of reliable methods of early detection and the poor prognosis associated with advanced ovarian cancer are the justifications for the performance of bilateral RRSO after completion of childbearing. Results of a meta-analysis involving 10 studies of carriers of a BRCA1/2 mutation showed an 85% reduction in the risk for ovarian or fallopian tube cancer following RRSO.^31^ The highest incidence rate for BRCA1 mutation carriers was observed between the ages of 50 and 59 years (annual risk, 1.7%); for BRCA2 mutation carriers, the highest incidence rate was observed between the ages of 60 and 69 years (annual risk, 0.6%).^32^ Performing an RRSO at the recommended ages (ages 35–40 for BRCA1 mutation carriers and at 40–45 years for BRCA2 mutation carriers) results in early menopause and the related effects and symptoms. In premenopausal women, RRSO (surgically induced menopause) results in early sterility and the risks associated with menopausal syndromes such as osteoporosis, cardiovascular disease, neuromotor and cognitive changes, changes to vasomotor symptoms, sexual concerns, and urogenital deficiency. The use of hormonal replacement therapy significantly compensates for hormonal deprivation and counteracts menopausal syndrome morbidity and mortality.^33^ Counseling a woman for RRSO should include a discussion and clarification of completion of childbearing desires, the extent of her personal cancer risk, the degree of protection for ovarian cancer, the impact it might have on quality of life, the management of menopausal symptoms, and the use of hormone replacement therapy. There have been studies that suggest a benefit of RRSO on breast cancer risk, but the significance and extent of the effect remains uncertain.^34^ Salpingectomy (removal of the fallopian tubes and fimbriae) alone has not been statistically proven as a method for risk reduction.^35^ The 2018 Cochrane study analyzed 10 reviews from the years 1999 through 2017 assessing the benefits and harms of RRSO in women with BRCA1 or BRCA2 mutations. In their review they compared RRSO versus no-RRSO in women without a previous or coexisting breast, ovarian, or fallopian tube malignancy, in women with or without hysterectomy, and in women with a risk-reducing mastectomy before, with, or after RRSO. The main outcomes of their analysis were that overall survival was longer with RRSO compared with no-RRSO (HR 0.32, 95% CI 0.19–0.54; *P*<0.001). High-grade serous ovarian cancer mortality (HR 0.06, 95% CI 0.02–0.17; I^2^=69%; *P*<0.0001) and breast cancer mortality (HR 0.58, 95% CI 0.39–0.88; I^2^=65%; *P*=0.009; 7 studies) were lower with RRSO compared with no-RRSO. The authors of the Cochrane review concluded that RRSO compared to no-RRSO in BRCA1 and BRCA2 mutation carriers, when analyzed together, showed an increase in overall survival. Nevertheless, when analyzed separately, there was a decrease in both high-grade serous cancer and breast cancer mortalities in BRCA1 mutation carriers, but not in BRCA2 mutation carriers. Data analysis from BRCA1 and BRCA2 mutation carriers together found no effect of RRSO together with risk-reducing mastectomy on breast cancer mortality.^28^ Clinical trials of interval salpingectomy and delayed oophorectomy are ongoing. The concern for risk-reducing salpingectomy alone is that individuals are still at risk for developing ovarian cancer.^36^

Risk-reducing surgeries are irreversible, and each has potential complications or harms. These may include bleeding, infection, anxiety and concerns about body image (bilateral risk-reducing mastectomy), and early menopause in premenopausal women (bilateral risk-reducing salpingectomy-oophorectomy). Some women have developed breast cancer, ovarian cancer, or primary peritoneal carcinomatosis (a type of cancer similar to ovarian cancer) even after risk-reducing surgery. Nevertheless, as mentioned earlier, these surgical procedures greatly reduce cancer risks.

## HALACHIC BACKGROUND

As a background to the issue at hand, we present a comprehensive halachic discourse, beginning with the fundamental principles relevant to saving lives and considering quality of life. The halachic issues relevant to BRCA screening and subsequent interventions are based upon the general halachic approach to health and healing. Most fundamentally, we are made in God’s image and have the ability and responsibility to be His partner in the creative process of bettering the world.^37^ Health professionals who unravel the mysteries of medicine and intervene to care and cure play a critical role in this process through their accountability for the optimal preservation of the precious commodity of life. In Exodus 21:19 the Bible stipulates *rapo y’rapeh* (thoroughly healed). Rashi (a renowned medieval commentator) and halachic authorities explain the repetition that appears in Hebrew: the root for the word “heal,” r-p-o, appears consecutively in two different forms). The purpose of this repetition is to disabuse us of a potentially erroneous notion: if God smites us with disease, healing is an act of rebellion.^38^

Saving lives (*pikuach nefesh*) overrides the Torah’s commandments as their very purpose is to sustain life. Leviticus 18:5 teaches: *U’Shmartem et chukotai v’et mishpatai … v’chai bahem* (Take heed to keep my laws … [you] shall live by them). The Babylonian Talmud adds: *v’lo sh’yamut bahem* (and not to die because of them).^39^ Rashi explains the reason for the addition: God values our lives more than His own commandments, and He therefore instructs us to set aside the latter for the former.^40^ The classic Talmudic case of *pikuach nefesh* is found in Tractate Yoma (84b): An avalanche occurred on the Sabbath, and it was not known if anyone was buried underneath the rubble and likewise not known if anyone underneath survived. The Talmud rules that we must set aside the sanctity of the Sabbath and dig into the rubble for the chance, however slim, of saving lives.

The Babylonian Talmud stipulates that if a life is threatened, one may not rely on a miracle (*ein somchin al hanes*), but must rather take action to avoid or eliminate the threat.^41^ From Tractate Ta’anit 20b we learn that it is forbidden to remain in a dangerous place and assume a miracle will come to the rescue. Even if a miracle occurs, continues the Talmud, this will be deducted from the individual’s “account of merits” as he neglected to do his share. The biblical source supporting this obligation is found in Deuteronomy 6:16: *Lo t’nasu et Hashem Elokeichem* (Don’t put the LORD your God to the test), as this would be a display of arrogance on man’s part.

Nevertheless, it must be acknowledged that normative living often entails unavoidable risks such as childbirth or professional hazards. The Babylonian Talmud legitimizes minimal risk-taking in order to fulfill fundamental commandments, such as performing a circumcision on a cloudy day (once thought to be dangerous), and cites Psalms 116:6.^42^ The verse in its entirety reads: *Shomer p’taim Hashem daloti v’li yhoshia* (God watches over the simple; I was brought low and He saved me), which Rabbi David Ben Kimchi understands to mean, “in situations in which I am unable to ward off danger, He saves me.”^43^ Various Responsa delineate caveats with respect to the legitimacy of risk-taking and relying on Divine providence, such as: the risk does not pose an immediate threat to the individual’s life;^44^ the risk has been demonstrated to be minimal or is so perceived by the public;^45^ all possible precautions have been taken;^46^ or there is an overriding communal or national concern which warrants special attention.^47^

### Screening as a Diagnostic Tool

Diagnosing and treating a life-threatening disease is halachically defined as *pikuach nefesh*. Therefore, it is logical that prevention or early detection via screening would be under the same category. Nevertheless, because the latter entails an additional element, it reveals a latent phenomenon, and an additional commandment may have relevance. Deuteronomy 18:13 teaches: *Tamim t’yeh im Hashem Elokecha* (Be wholehearted [blameless] with the LORD, your God). Rashi explains: do not attempt to predict the future but rather accept what comes with full trust in the Divine. Rashi and other commentators^48^ understand the application of the commandment in its context, as it immediately follows the prohibition of seeking out soothsayers and fortune tellers to predict the future. Rabbi Yechiel Epstein stipulates that *tamim t’yeh* denotes an obligation to seek healing only from reputable physicians, albeit, he stipulates, together with prayer and good deeds.^49^

A preoccupation with screening as a vehicle to unmask all of one’s maladies could be considered a violation of *tamim t’yeh.*^50^ Given the current state of medical science, this is an unrealistic goal, and therefore, in this regard, we need to remain *tamim* (wholehearted), putting our trust in God. Rabbi Yitzchak Zilberstein, a renowned contemporary halachic authority, goes a step further, maintaining that ultrasound screening for fetal defects, without an index of suspicion for a specific finding, is at odds with *tamim t’yeh*.^51^ There are, however, halachic authorities who disagree with his position; they maintain that the safety, accessibility, affordability, and accuracy of fetal ultrasound render its findings as virtually known. These characteristics, coupled with the potential life-saving interventions currently available, put this type of screening in the category of *pikuach nefesh*.^50^

Rabbi Moshe Feinstein, a prominent twentieth-century halachic authority, discusses genetic screening for Tay–Sachs disease in light of *tamim t’yeh.* He rules that the ease and accuracy of the test, together with the severity of the disease and the ability to prevent it, are halachic grounds for its uptake. He further stipulates that not screening for Tay–Sachs is equivalent to closing one’s eyes to something that is open to view and therefore not a violation of *tamim t’yeh*.^52^ Rabbi Yitzchak Zilberstein supports Tay–Sachs screening specifically, for similar reasons.^53^ Rabbi David Bleich maintains that refraining from the recommended screening for Tay–Sachs in the Jewish Ashkenazi community is a violation of *tamim t’yeh* because it is a rejection of God’s providence embodied in the knowledge He gave man to engineer this effective screening process.^54^

Rabbi Menasseh HaKatan, on the other hand, claims that Tay–Sachs screening is a violation of *tamim t’yeh.* He argues that matches are made in heaven, and we are not permitted to tamper with the delicate complex process through which two individuals decide to build their lives together, by making their union conditional on screening results for a rare illness. Rabbi HaKatan maintains that if we cannot eliminate all the myriads of risks involved in bringing children to the world, we are not obligated to single out the rare risk of Tay–Sachs. He extrapolates this conclusion from a ruling regarding the presence of anatomical defects in animals which render them unkosher. If it is not feasible to check for all anatomical defects, one is not obligated to check for those that are not common. He does not address the principle of *Sakanta Chamirah Mi’Isura* (Babylonian Talmud, Tractate Chulin 10a) (danger to life and health requires more stringent vigilance than ritual prohibitions). Rabbi HaKatan further rules that screening is forbidden because it can be detrimental as it may cause the breakup of an engagement.^55^ It is important to point out that the presence of the defective gene in both potential partners is most often ascertained before two individuals become emotionally involved. Screening for Tay–Sachs has in fact gained almost universal halachic support in all the sectors of the Jewish observant community, including that of the ultraorthodox.

### Screening for BRCA Mutations

Children inheriting the Tay–Sachs gene from both parents will inevitably develop this incurable disease and die during the first years of life; however, BRCA mutation carriers and their carrier children do not necessarily develop cancer–although many will do so over their lifetime. Like screening for Tay–Sachs, BRCA screening involves an accessible, safe, and simple blood test and is highly accurate. There are, furthermore, effective risk-reducing interventions available for individuals identified as mutation carriers and the option of preimplantation genetic diagnosis for preventing the mutation from being passed on to the next generation. Considering these advantages and the relatively high prevalence of BRCA mutations in the Jewish Ashkenazi population, the Israeli Ministry of Health (as of 2020) recommends and finances screening for all women of this ethnic descent, regardless of family history (the latter has been found to be an unreliable criterion, missing about 50% of the carriers).^56^ Guidelines of the Ministry of Health are considered by halacha as reflecting *refuah b’duka* (evidence-based medical practice); barring the presence of halachic issues, adherence is obligatory for the observant Jewish population.^57^ In matters of *pikuach nefesh*, adherence would be so even if commandments would need to be overridden.

To qualify for *pikuach nefesh* status, the threat to life for a specific individual or a group of individuals must be present, not just possible at a future time.^58^ This is referred to by the *Chatam Sofer* and other halachic authorities of modern times as *hacholeh l*’*fanainu* (the unwell individual is before us), someone whose life is being threatened and can potentially be rescued because they are right here, in front of us.^59^ The *Chazon Ish* defines *pikuach nefesh* as a situation in which it is known that the threat is present even though the individual being threatened is not literally in front of us (for example, in an epidemic).^60^ Other authorities, in a similar vein, maintain that if the seeds for a life-threatening situation have already been sown, this is to be considered *pikuach nefesh.*^61^ Rabbi Eliezer Waldenberg, a prominent halachic authority of the previous generation, clarifies the ruling by setting two criteria for *pikuach nefesh*: (1) a concrete threat, and (2) a specific individual (or individuals) being threatened at the present time. He maintains, however, that even in situations in which threat to life will only materialize at a future time, if the chances of this occurring are significant, they must be treated as *pikuach nefesh* in the present.^62^ Similarly other authorities make the distinction between immediate *pikuach nefesh* which warrants action even if the risk to life is minimal, and future *pikuach nefesh* which requires action (also overriding commandments) specifically if the risk is significant.^61^

An additional criterion for *pikuach nefesh*, for the purpose of overriding commandments, is that the threat to life must not be negligible. Some authorities set a one in a thousand chance (of the existence of the threat or the feasibility of the rescue) as the minimum, while others designate a range of 0.5%–5% chance.^63^ Some set subjective criteria maintaining that threat to life for the purposes of *pikuach nefesh* is what the public or the medical community perceives it to be.^63^,^64^

Rabbi Bakshi-Doron maintains that BRCA mutation carrier status should be categorized as a *pikuach nefesh*, given that the seeds for serious disease have already been sown and the lifetime risks of developing cancer are over 50%. He further considers screening for BRCA as a command although not obligatory because the available interventions do not totally prevent the disease.^65^

Rabbi Shlomo Yosef Elyashiv, a prominent halachic authority of the previous generation, rules that BRCA screening is not categorically obligatory but recommends that it should be individually discussed with a halachic authority. His reservations are the low statistical risk (2.5%) of being a mutation carrier coupled with the fact that not all carriers develop disease. Additional issues for this halachic authority are the unavailability of optimal risk-reducing interventions, the potential for anxiety related to carrier status, and the quandaries of how to deal with the information vis-à-vis the family and social contacts.^66^ The two last-mentioned considerations are of import as mental anguish is halachically recognized as an element of ill health. Exodus 21, verses 18–25 specifically stipulate that one who injures his fellow man is obligated to compensate for the disability and medical expenses, incurred loss of employment, and physical pain. The nuances of the original text, however, also imply an obligation to compensate the injured party for the mental anguish and humiliation they have experienced. The Babylonian Talmud enumerates *boshet* (humiliation) as requiring compensation.^67^ Rabbi Yitzchak Zilberstein concurs with Rabbi Elyashiv’s reasons for limiting the obligation to screening; he nevertheless recommends that individuals of Ashkenazi descent who have a positive family history should screen for BRCA mutations.^66^^(p96,fn146)^ However, current research demonstrates that limiting screening in this manner actually misses 50% of BRCA carriers and it is no longer considered a reliable risk criterion by the medical community.

Among the rabbinical authorities who obligate screening, there are differences of opinion with respect to its optimal timetable. Rabbi Bakshi-Doron in Responsa Binyan Av 5:65 designates post childbearing as the desirable time to screen for BRCA mutations. His reasoning is that the recommended risk-reducing surgical removal of the ovaries is not halachically permitted before that time. Knowing one is a BRCA mutation carrier without the ability to avail oneself of this intervention could be anxiety-provoking. Rabbi Professor Steinberg considers age 25–30 as the most appropriate time for screening as many individuals are married by then and the risk for breast cancer rises significantly after this age.^66^^(p96,fn149)^ Rabbi Abargil recommends that women should wait to screen until they are married, to avoid the stigma which can affect finding a suitable marriage partner.^66^^(p96,fn150)^ Rabbi Neventzal maintains that it should be done before marriage in order to avoid casting doubt on the halachic status of the marriage (had one of the partners known about the other being a carrier, they might not have entered into the marriage).^66^^(p96,fn151)^ The difference of opinion among the authorities perhaps opens a legitimate halachic window for choosing different time periods.

## HALACHIC CONSIDERATIONS

### Risk-reducing Interventions for Breast Cancer

Intervening to reduce breast cancer risk is halachically obligatory as it is *pikuach nefesh*; two interventional options are recognized by the medical community. The first, vigilant surveillance via MRI, mammography, breast ultrasound, and frequent breast examinations are successful in early detection but not for breast cancer prevention; they are, however, non-invasive and considered to be risk-free. On the other hand, they might not relieve the anxiety of developing the disease. The second, bilateral mastectomy, is effective in reducing the risk of the cancer occurrence. However, it entails major surgery with a concomitant albeit relatively low risk for mortality and morbidity. Possible psychological ramifications of surgery and its aftermath may involve body image issues and strains in marital intimacy; these factors carry halachic weight. More broadly, quality of life is an important halachic consideration, as serving God can be optimally accomplished only if an individual enjoys overall well-being.^68^ The sages equate wisdom with the commandments of Torah.^69^ From that perspective Torah’s commandments can be understood as the *darchai noam* (pleasant paths) referred to in Proverbs 3:17. They were given to us, not only to sustain life itself, but to render life meaningful, enjoyable, and devoid of suffering.^70^ In addition to acknowledging the value of quality of life in its own right, halachic authorities acknowledge that poor quality of life can pose a threat to health and life itself. This finds expression in the Babylonian Talmud, which takes a lenient stand in order to avoid rendering a woman an aguna (literally, a “chained woman). Such a status would leave her devoid of a socio-economic support system.^71^ Rashi explicates: consideration is given to the danger of leaving her devoid of a partner. In his Responsa, Rabbi Yehoshua Aharonberg, based upon Rashi’s reasoning and his analysis of a previous responsa, concludes that being an *aguna* can have a detrimental effect on the woman’s psychological health, which could trigger a life-threatening illness.^72^ However, quality of life is often subjectively determined. Proverbs 14:10 teaches: *Lev yodea marat nafsho* (The heart knows its own bitterness); in other words, one’s own heart is best attuned to ascertaining one’s needs.^73^ As individuals will differ regarding which intervention will give them optimal quality of life along with longevity, their perceptions have halachic import.

There are two additional halachic issues that are pertinent to risk-reducing mastectomy. *The first* relates to the risk of the surgery. There is broad halachic consensus that an individual suffering from a disease shortening his life expectancy to less than a year is permitted to undergo a medical procedure which, although risky, gives him/her a chance of a normal life expectancy.^74^,^75^ The Talmud teaches that, in a situation in which there is an intervention that has the chance of giving long-term survival, its risks are not to be taken into account.^76^ There are, however, differences of opinion regarding what degree of risk may be incurred, for what degree of chance for a normative span. Some authorities maintain that it is permitted (but not obligatory) to take a risk even if one currently has a life expectancy of several years for the chance of a yet longer life expectancy. In this case it is the individual’s responsibility to determine what option is most beneficial for him/her.^77^ These rulings, however, are not fully applicable to a risk-reducing mastectomy because the BRCA carrier does not yet have cancer and might not develop the disease at all.

There is an additional ruling that might have halachic relevance. Rabbi Yisrael Lipshutz rules that when faced with two risky situations, an individual should choose the option with the least risk. He utilizes this principle to obligate individuals to embrace the low risk of inoculation against smallpox (discovered in his lifetime) in order to ward off the much higher risk of contracting the life-threatening virus.^78^ This situation is similar to BRCA in that the individual has not yet been infected and might never be. On the other hand, smallpox cannot be detected early by surveillance and inoculation is the only risk-reducing option. *The second* issue is the prohibition of inflicting injury on oneself. Most halachic authorities are of the opinion that this prohibition does not pertain if the injury is not done to harm or humiliate, but is rather undertaken for the individual’s benefit, which is the case with respect to mastectomy.^79^,^80^

To the best of our knowledge, Rabbi Asher Weiss is the only authority that has written a specific halachic opinion regarding breast cancer risk-reducing interventions. In response to a woman who requested permission for a mastectomy after four of her siblings died of breast cancer, Rabbi Asher Weiss acknowledges that there is no absolute halachic prohibition against opting for the surgery, and he leaves the decision up to the woman. He does, however, express his own preference for strict surveillance coupled with remaining *tamim*, that is, trusting in God’s providence.^81^ In an oral communication with Rabbi Professor Avraham Steinberg, Rabbi Zalman Nechemia Goldberg also granted permission for the risk-reducing mastectomy option.^82^

### Risk-reducing Interventions for Ovarian Cancer

Risk-reducing salpingectomy-oophorectomy (RRSO) is the medical intervention of choice for prevention of ovarian cancer. Available surveillance methods for early ovarian cancer detection (i.e. gynecological ultrasound and tumor blood markers) have been shown not to be effective; in most cases ovarian cancer is discovered at a late stage, making the survival rate poor. As pointed out in the scientific section, removal of the ovaries with the fallopian tubes reduces the risk of contracting ovarian cancer by over 90% and substantially decreases all-cause mortality by 68%.^9^ Current research, however, indicates that surgically induced menopause can have detrimental repercussions on a woman’s health. Risks for cognitive decline, heart disease, and osteoporosis are all significantly higher compared to those occurring after natural menopause, and risks go up in tandem with earlier surgical age.^33^ These risks have nevertheless not impacted medical experts’ recommendation for RRSO as the state-of-the-art risk-reducing measure for BRCA mutation carriers. Ovarian cancer with poor survival rate is still the greater concern, and hormone replacement therapy has been successful in warding off its detrimental effects. It is of much import halachically that the risk for ovarian cancer becomes significant after 35–40 for BRCA1 mutation carriers and 5–10 years later for BRCA2 mutation carriers.

Salpingectomy-oophorectomy is at odds with the command of *p’ru u’rvu* (be fruitful and multiply), a fundamental commandment which is also critical to the survival of the Jewish people. Halachic authorities generally rule that women should wait with surgery until completing childbearing (also recommended by international guidelines).^66^^(p92,fn128,130)^ The literature reports that pregnancies minimize the risk for ovarian cancer because they decrease the number of lifetime ovulations.^83^ Rabbi Bakshi Doron (Binyan Av 5: 63, 65) encourages but does not obligate women to have large families. He rules that a woman is obligated to postpone surgery until she has one child of each gender, although Rabbi Feinstein just stipulates “children.”^84^ The goal of establishing a family justifies the small risk of contracting ovarian cancer at a young age. The additional halachic issue relevant for RRSO is the prohibition of *seirus* (castration). Most halachic authorities delineate this prohibition to be of rabbinic rather than biblical origin with respect to women. Therefore, this prohibition must be overridden even according to authorities who define BRCA-related cancers as a future and uncertain risk rather than a certain and immediate one. Neverthess, for the minority of authorities who rule *seirus* of women to be of biblical origin, this prohibition can also be overridden as it is a case of *pikuach nefesh*, since the risk of BRCA-related ovarian cancer is increased by more than 50%.^65^

## SUMMATION

Saving lives and preserving quality of life are perceived by halacha as sacred goals. The optimal course of action to achieve these goals with respect to BRCA screening and interventions is often not definitive and varies among individuals. Halacha encourages the exercise of informed autonomy in decision-making when objective beneficence is not clear-cut. We are the ultimate wardens of our lives and have unique insight into what is most beneficial for ourselves. This is not the classic autonomy celebrated by general ethicists because observant Jews are committed to halacha as their guiding code. It is halacha which authorizes autonomy, not as a right but as a duty.

As the issues surrounding BRCA are complex and vary among individuals turning to counseling, the input of health professionals is critical. They need to empower those they care for with the knowledge necessary for optimal decision-making. Professionals providing counseling regarding the issue of BRCA mutations are often consulted before the halachic authorities. Health professionals provide those they counsel with the pertinent information for decision-making and assist them in formulating targeted questions for their Rabbis. To do so, they themselves need to be well versed in the relevant halachic issues. To this end we have undertaken our discourse.

## Abbreviations

BRCA: breast cancer gene
MRI: magnetic resonance imaging
RRBM: risk-reducing bilateral mastectomy
RRSO: risk-reducing salpingectomy-oophorectomy
